# Prognostic value of immunosuppression scores in patients with esophageal squamous cell carcinoma: a multicenter study

**DOI:** 10.3389/fimmu.2024.1517968

**Published:** 2025-01-07

**Authors:** Shao-jun Xu, Yun-fan Luo, Jin Huang, Jia-hua Tu, Chao Chen, Yan-ming Shen, Zhao-min Sun, Shu-chen Chen

**Affiliations:** ^1^ Department of Thoracic Surgery, Fujian Medical University Union Hospital, Fuzhou, Fujian, China; ^2^ Key Laboratory of Ministry of Education for Gastrointestinal Cancer, Fujian Medical University, Fuzhou, Fujian, China; ^3^ Key Laboratory of Cardio-Thoracic Surgery (Fujian Medical University), Fujian Province University, Fuzhou, Fujian, China; ^4^ Department of Thoracic Surgery, The First Hospital of Putian, Putian, Fujian, China

**Keywords:** esophageal squamous cell carcinoma (ESCC), human leukocyte antigen-E (HLA-E), natural killer (NK) cells, inverse probability treatment weighting (IPTW), prognosis

## Abstract

**Introduction:**

The prognostic impact of human leukocyte antigen-E (HLA-E) expression and the proportion of natural killer (NK) cells in esophageal squamous cell carcinoma (ESCC) was investigated.

**Methods:**

This study retrospectively evaluated 397 ESCC patients across two centers. The cumulative incidence of recurrence (CIR) and the incidence of tumor-related death (CID) were analyzed in various groups. An immunosuppression score (ISS) was developed based on HLA-E expression and NK cell proportion. Differences between groups were adjusted using inverse probability treatment weighting (IPTW). The factors influencing cancer-specific survival (CSS) and recurrence-free survival (RFS) were also examined.

**Results:**

Patients with low HLA-E expression had significantly higher five-year CIR and CID compared to those with high expression (CIR: 20.7% vs. 45.1%, CID: 19.3% vs. 40.1%; *p*< 0.001). Similarly, NK cell-positive patients had significantly better five-year CIR and CID than NK cell-negative patients (CIR: 16.3% vs. 59.6%, CID: 13.9% vs. 53.7%; *p* < 0.001). The Sankey diagram indicated that the low ISS group had a lower recurrence and tumor-related mortality rate (*p* < 0.05). After IPTW adjustment, the low ISS group showed improved five-year RFS (80.1% vs. 35.4%, *p* < 0.001) and five-year CSS (82.3% vs. 42.5%, *p* < 0.001) compared to the high ISS group.

**Conclusions:**

ESCC with different ISS statuses represents two distinct biological subtypes, underscoring the need for personalized treatment strategies tailored to varying tumor behaviors.

## Introduction

Esophageal squamous cell carcinoma (ESCC) is the most prevalent form of esophageal cancer in Asia, accounting for approximately 90% of cases (1, 2). In recent years, immune checkpoint blockade (ICB) immunotherapy has shown promising results in advanced ESCC, but its efficacy remains limited, with objective response rates ranging from 6.4% to 33.3% (3–5). Therefore, identifying novel immune checkpoint biomarkers and developing more effective immunotherapeutic strategies is crucial.

Human leukocyte antigen-E (HLA-E), a non-classical class I HLA molecule, is widely expressed on nucleated cells and has been associated with prognosis in various malignancies (6–8). HLA-E interacts with NK Group 2 member A (NKG2A) on natural killer (NK) cells, inhibiting cytotoxic activity and facilitating immune evasion by tumors (9, 10). NKG2A acts as an inhibitory checkpoint receptor, and blocking the NKG2A/HLA-E axis has been shown to enhance anti-tumor immune responses by increasing NK cell activity (11, 12).

Moreover, NK cells are a key component of the innate immune system and play a critical role in the surveillance and elimination of tumor cells (13–15). In gastric and colorectal cancers, NK cell levels serve as sensitive prognostic markers, with higher NK cell infiltration associated with a more favorable prognosis (16–18). Studies have suggested that the combined assessment of HLA-E expression in tumors, immune cells, and antigen-presenting cells, along with NK cell levels, could provide an effective prognostic tool for advanced gastric cancer patients (19). However, the potential of combining NK cell and HLA-E levels to predict clinical outcomes in ESCC remains unclear.

This study utilized various immunofluorescence techniques to examine the relationship between HLA-E levels and NK cell proportion in ESCC. Additionally, an immunosuppression score (ISS) was proposed based on both NK cell proportion and HLA-E expression. To the best of our knowledge, this is the first investigation to explore the prognostic potential of combining HLA-E expression and NK cell status after surgical resection of ESCC.

## Materials and methods

### Patients

This research included 397 diagnosed ESCC patients who enrolled at Fujian Medical University Union Hospital (n = 299) and The First Hospital of Putian (n = 98) from January 2011 to December 2016. Patients who (1) had histopathologically confirmed ESCC; (2) fell into the I-IVA stages defined by the American Joint Committee on Cancer (AJCC; 8th edition); (3) had not undergone neoadjuvant chemoradiotherapy before surgery; (4) had no other type of malignancy; (5) had no distance were selected for this research. Exclusion criteria: (1) patients with incomplete clinicopathological data or missing follow-up; (2) those who died within 30 days after surgery. This investigation was authorized by the Ethics Committee of the institution (No. 2021KJCX068).

### Multiplex immunofluorescence

Following formalin-fixation and paraffin-embedding, tissue section (4 µm) were dewaxed and rehydrated with gradient ethanol. Then, after antigen repairing, the activity of endogenous peroxidase was quenched with 0.3% H_2_O_2_ and the tissues were blocked using 5% goat serum. The samples were tagged with rabbit anti-human HLA-E (Abcam, ab203082, 1:2000) antibodies and with NK cells using rabbit anti-human CD56 (Abcam, ab119587, 1:2000) and CD3 (Abcam, ab16669, 1:2000) antibodies. Subsequently, the sections were treated with the corresponding horseradish peroxidase (HRP) conjugate secondary antibody and with DAPI to stain the nucleus. After quenching of self-fluorescence, the sections were sealed, and evaluated and imaged under fluorescence microscopy.

### Multiplex immunofluorescence analysis

In evaluating the positive signal intensity (SI) of HLA-E expression, we employ a more objective quantification method. We use a digital pathology slide scanner to perform high-resolution, high-quality digital scanning of tissue sections. Then, the images are automatically analyzed using Aipathwell, an AI-based digital pathology image analysis software developed by Servicebio (20). The specific process is as follows: first, mthe system automatically locates and delineates the region of interest; next, the SI is automatically calculated based on the HIS (Hue-Saturation-Intensity) model; finally, the SI is classified according to the calculated results. This process can be manually adjusted as needed to ensure the accuracy of the results.

In evaluating the positive or negative expression of CD56, we also use Aipathwell, Aipathwell utilizes deep learning principles and algorithm training based on vast amounts of data to build an efficient automated image analysis system. In this system, if the positive SI of CD56 is graded as 0, it is classified as negative; if the SI is graded ≥1, it is classified as positive.

### HLA-E expression and NK cell proportion

The semi-quantitative immunoreactive score (IRS) of HLA-E was evaluated. IRS = positive intensity (SI)× positive cell ratio (PP) (21). The SI was divided into the following grades: 0, no staining; 1, weak positive/light yellow; 2, medium positive/brown-yellow; 3, strong positive/brown. Moreover, PP levels included: 0 = 0 ~ 5%, 1 = 6 ~ 25%, 2 = 26 ~ 50%, 3 = 51 ~ 75%, and 4 = > 76% (19). The IRS has an overall score of 0-12.

Ten high-power (10×40) fields were randomly selected to measure the number of NK cells in tumor nests. CD56 positive and CD3 negative staining identified NK cells. NK cell proportion = number of NK cells/total number of cells.

### Definition of concept

**Figure 1:**
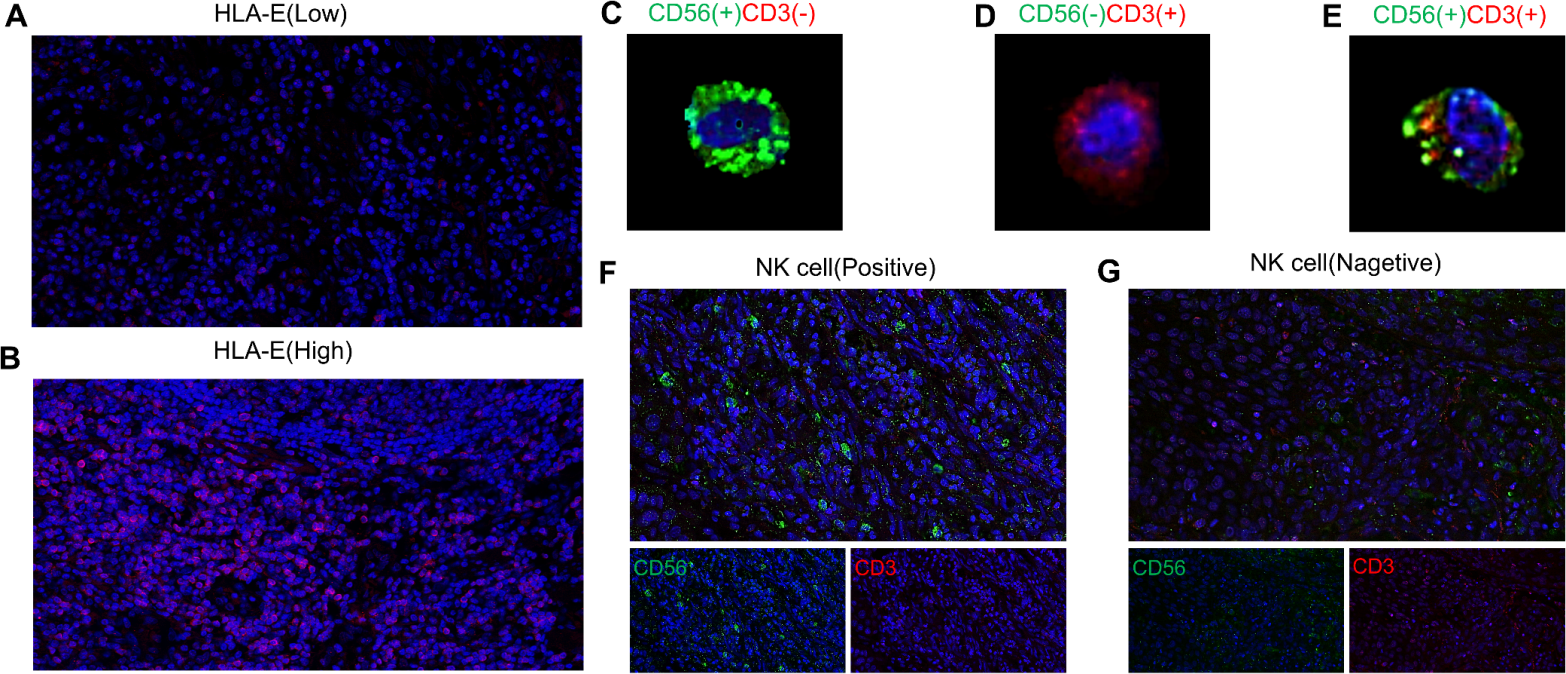
Expression of human leukocyte antigen E [low expression **(A)**; high expression **(B)**] and NK cells(the NK cells exhibit CD3 negative and CD56 positive staining **(C)**, whereas CD56 negative and CD3 positive **(D)** or CD56 positive and CD3 positive **(E)** are not associated with NK cells) in ESCC [positive **(F)** negative **(G)**].

According to the median HLA-E score (**Supplementary Figure S1A**), IRS < 6 = low expression of HLA-E and IRS ≥ 6 = high expression of HLA-E (**Figures 1A, B**). NK cells exhibit CD3 negative and CD56 positive staining (**Figure 1C**), whereas CD56 negative and CD3 positive (**Figure 1D**) or CD56 positive and CD3 positive (**Figure 1E**) are not associated with NK cells. Using the median NK cell proportion (**Supplementary Figure S1B**), NK cells ≥ 4.18% = positive (**Figure 1F**) and NK cells < 4.18% = negative (**Figure 1G**).

### Follow-up analysis

Post-surgery, patients were followed up every three months for the first year, every six months for the subsequent five years, and annually thereafter until either death or the cutoff date of December 2019. Recurrence-free survival (RFS) was defined as the time from surgery to the occurrence of tumor recurrence, metastasis, or death. Cancer-specific survival (CSS) was determined from the days between surgery and death due to the tumor or the end of the last follow-up.

### Statistical analysis

For intergroup categorical variables, Fisher exact or χ2 tests was applied with t-tests or Mann-Whitney U tests for continuous variables. The cumulative incidence curve compared the influence of HLA-E level and NK cell proportion on cumulative incidence of recurrence (CIR) and cumulative incidence of tumor-related death (CID). The receiver operating characteristic (ROC) area under the curve (AUC) was used for comparing predictive capabilities. Associations between mortality, recurrence rates, and the HLA-E-NK cell interaction with immunosuppressive scores (ISSs) were analyzed by Sankey diagrams.

Inverse probability treatment weighting (IPTW) is used to balance the intergroup differences, and the standardized mean difference (SMD) < 0.1 is considered a variable balance. IPTW for low ISS is calculated as 1/propensity score (PS), and IPTW for high ISS is calculated as 1/(1-PS). The Kaplan-Meier test was employed to elucidate patients’ RFS and CSS. Furthermore, with the help of the log-rank test, the intergroup survival differences were determined. Univariate and multifactorial analyses were performed with Cox regression to elucidate independent risk factors affecting patients with ESCC. All analyses were bilateral, and p-values <0.05 were termed significant. Both SPSS v26.0 and R v3.6.3 were applied for analyses.

## Results

### Clinicopathological characteristics of different groups

**Table 1** demonstrates the clinicopathological features of different groups. High HLA-E expression patients had higher N stages (*p = 0.039*) and included more males (*p = 0.027*) than low HLA-E expression patients. No marked variations were seen in terms of age (*p = 0.405*), tumor location (*p = 0.154*), BMI (*p = 0.241*), histological grade (*p = 0.076*), T stage (*p = 0.145*), lymphadenectomy (*p = 0.269*), TNM stage (*p = 0.251*), and operative procedure (*p = 0.391*).

**Table 1:** Clinical characteristics of different HLA-E expression and NK cell status.

Characteristic	HLA-E		P-value	NK cell		P-value
	High	Low		Positive	Negative	
	n=257	n=140		n=209	n=188	
Sex			0.027			0.503
Female	55 (21.4%)	44 (31.4%)		55 (26.3%)	44 (23.4%)	
Male	202 (78.6%)	96 (68.6%)		154 (73.7%)	144 (76.6%)	
Age			0.405			0.87
≤65	208 (80.9%)	118 (84.3%)		171 (81.8%)	155 (82.4%)	
>65	49 (19.1%)	22 (15.7%)		38 (18.2%)	33 (17.6%)	
BMI (kg/m2)			0.241			0.151
≤18.5	30 (11.7%)	13 (9.3%)		23 (11.0%)	20 (10.6%)	
18.5-25	176 (68.5%)	107 (76.4%)		156 (74.6%)	127 (67.6%)	
≥25	51 (19.8%)	20 (14.3%)		30 (14.4%)	41 (21.8%)	
Histologic grade			0.076			0.057
Gx/G1	116 (45.1%)	48 (34.3%)		79 (37.8%)	85 (45.2%)	
G2	122 (47.5%)	83 (59.3%)		119 (56.9%)	86 (45.7%)	
G3	19 (7.4%)	9(6.4%)		11 (5.3%)	17 (9.0%)	
Tumor location			0.154			0.194
Proximal	20 (7.8%)	11 (7.9%)		19 (9.1%)	12 (6.4%)	
Mid	157 (61.1%)	98 (70.0%)		139 (66.5%)	116 (61.7%)	
Distal	80 (31.1%)	31 (22.1%)		51 (24.4%)	60 (31.9%)	
T stage			0.145			0.233
T1	58 (22.6%)	40 (28.6%)		60 (28.7%)	38 (20.2%)	
T2	56 (21.8%)	18 (12.9%)		37 (17.7%)	37 (19.7%)	
T3	137 (53.3%)	78 (55.7%)		106 (50.7%)	109 (58.0%)	
T4a	6(2.3%)	4(2.9%)		6(2.9%)	4(2.1%)	
N stage			0.039			0.001
N0	117 (45.5%)	73 (52.1%)		110 (52.6%)	80 (42.6%)	
N1	66 (25.7%)	43 (30.7%)		64 (30.6%)	45 (23.9%)	
N2	58 (22.6%)	22 (15.7%)		31 (14.8%)	49 (26.1%)	
N3	16 (6.2%)	2(1.4%)		4(1.9%)	14 (7.4%)	
TNM stage			0.251			0.038
I	60 (23.3%)	34 (24.3%)		52 (24.9%)	42 (22.3%)	
II	73 (28.4%)	45 (32.1%)		72 (34.4%)	46 (24.5%)	
III	110 (42.8%)	59 (42.1%)		80 (38.3%)	89 (47.3%)	
IVA	14 (5.4%)	2(1.4%)		5(2.4%)	11 (5.9%)	
Lymphadenectomy			0.269			0.073
Two-field	205 (79.8%)	118 (84.3%)		177 (84.7%)	146 (77.7%)	
Three-field	52 (20.2%)	22 (15.7%)		32 (15.3%)	42 (22.3%)	
Surgical procedure			0.391			0.731
McKeown	230 (89.5%)	129 (92.1%)		190 (90.9%)	169 (89.9%)	
Ivor Lewis	27 (10.5%)	11 (7.9%)		19 (9.1%)	19 (10.1%)	

Cases with NK-cell positivity had lower TNM (*p = 0.038*) and N stage (*p = 0.001*) than negative cases. Sex (*p = 0.503*), age (*p = 0.870*), BMI (*p = 0.151*), histological grade (*p = 0.057*), tumor location (*p = 0.194*), T stage (*p = 0.233*), lymphadenectomy (*p = 0.073*) and operative procedure (*p = 0.731*) were similar in both groups.

### Cumulative incidence curve

**Figure 2:**
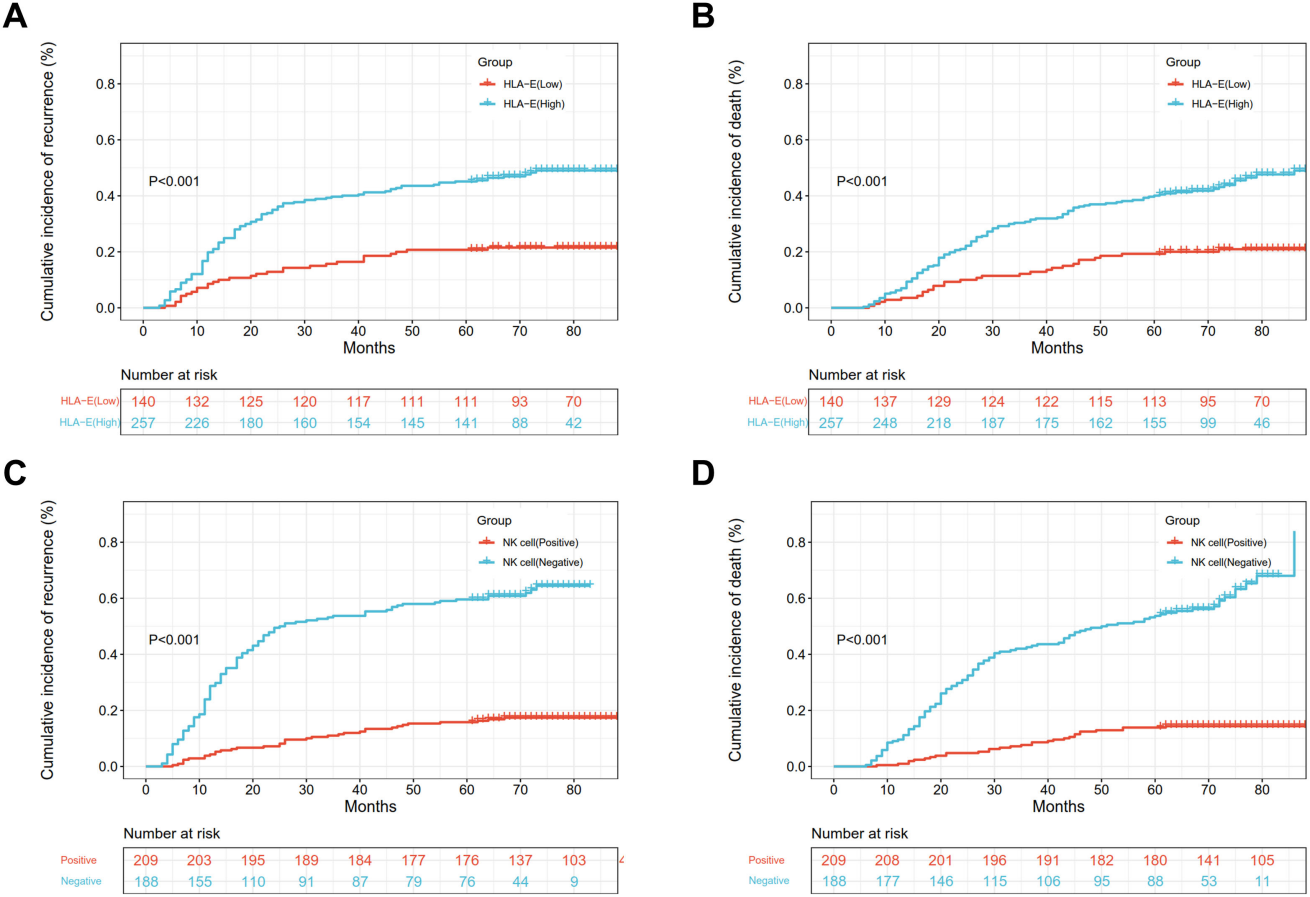
The CIR and CID of different HLA-E **(A, B)** and NK cell expressions **(C, D)** in ESCC patients.

The five-year CIR was 45.1% in patients with high HLA-E and 20.7% in those with low HLA-E (*p < 0.001*) (**Figure 2A**), while the five-year CID values were 40.1 and 19.3%, respectively (*p < 0.001*) (**Figure 2B**). Multivariate analysis showed an association between high HLA-E and increased CIR [95% confidence interval (CI): 1.291 - 3.000, hazard ratio (HR) = 1.968, *p = 0.002*] and CID (95% CI: 1.126 - 2.643, HR = 1.725, *p = 0.012*) (**Supplementary Table S1**).

The five-year CIR (**Figure 2C**) were 16.3 and 59.6% in the NK cell-positive and -negative groups, and the differences were significant (*p < 0.001*). Furthermore, the five-year CID was 13.9 and 53.7% in the NK cell-positive and -negative groups (**Figure 2D**), and the differences were significant (*p < 0.001*). Multivariate analysis indicated that the negative status of NK cells was linked with increased CIR (95% CI: 3.106 - 6.832, HR = 4.606, *p < 0.001*) and CID (95% CI: 3.693 - 8.739, HR = 5.681, *p < 0.001*) (**Supplementary Table S1**).

### The ISS was established based on the HLA-E expression and NK cell status

According to Cox multivariate regression analysis, low HLA-E expression = 0 score, high HLA-E expression = 1 score, negative NK cell = 1 score, and positive NK cell = 0 score. The overall score of 0 or 1 was defined as a low ISS, whereas a score of 2 indicated a high ISS (**Supplementary Figure S2**).

The AUC showed that the ISS was higher than the expression of HLA-E and NK cell status alone in predicting patient recurrence (ISS: AUC = 0.747; HLA-E: AUC = 0.639; NK cell: AUC = 0.730) (**Supplementary Figure S3A**). Additionally, ISS was also higher than the HLA-E level and NK cell status in predicting tumor-related death in patients (ISS: AUC = 0.763; HLA-E: AUC = 0.632; NK cell: AUC = 0.745) (**Supplementary Figure S3B**).

### Dynamically analyzed the effects of ISS on patient’s recurrence and tumor-related death by Sankey diagram

The Sankey diagram dynamically indicated the relationship of ISS with patient’s recurrence and tumor-related death. In the low ISS group, 51 (20.5%) patients indicated recurrences, whereas in the high ISS group, 102 (68.9%) patients had recurrences, and the differences in the recurrence rate were significant (*p < 0.001*).

**Figure 3:**
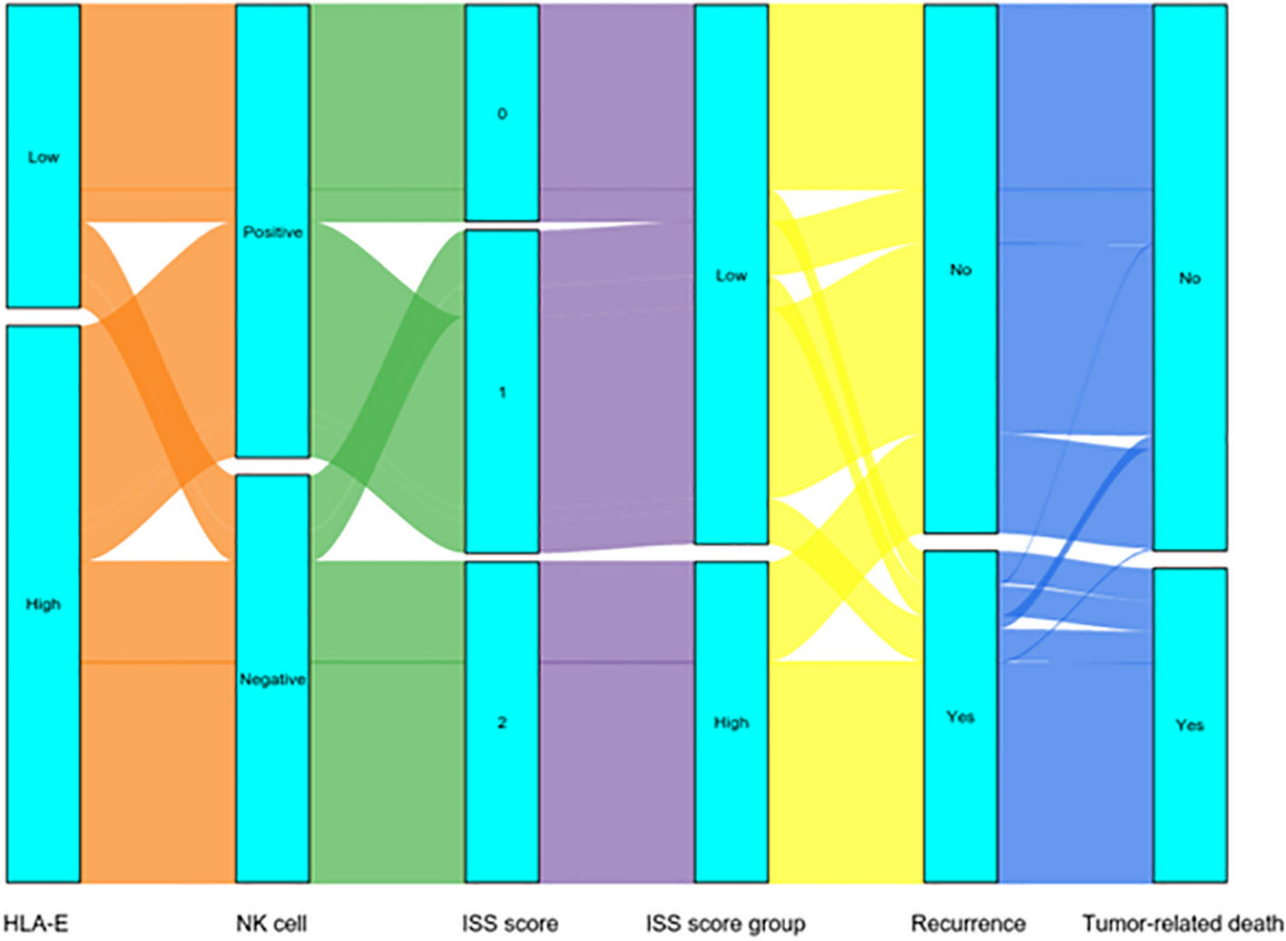
Sankey diagrams shows recurrence and tumor-related death with different ISS.

In terms of survival, there were 44 (17.7%) patients had tumor-related deaths in the low ISS group, and 101 (68.2%) patients had tumor-related deaths in the high ISS group, and the differences were significant (*p < 0.001*) (**Figure 3**).

### The association of IPTW balance ISS with clinicopathological factors

**Figure 4:**
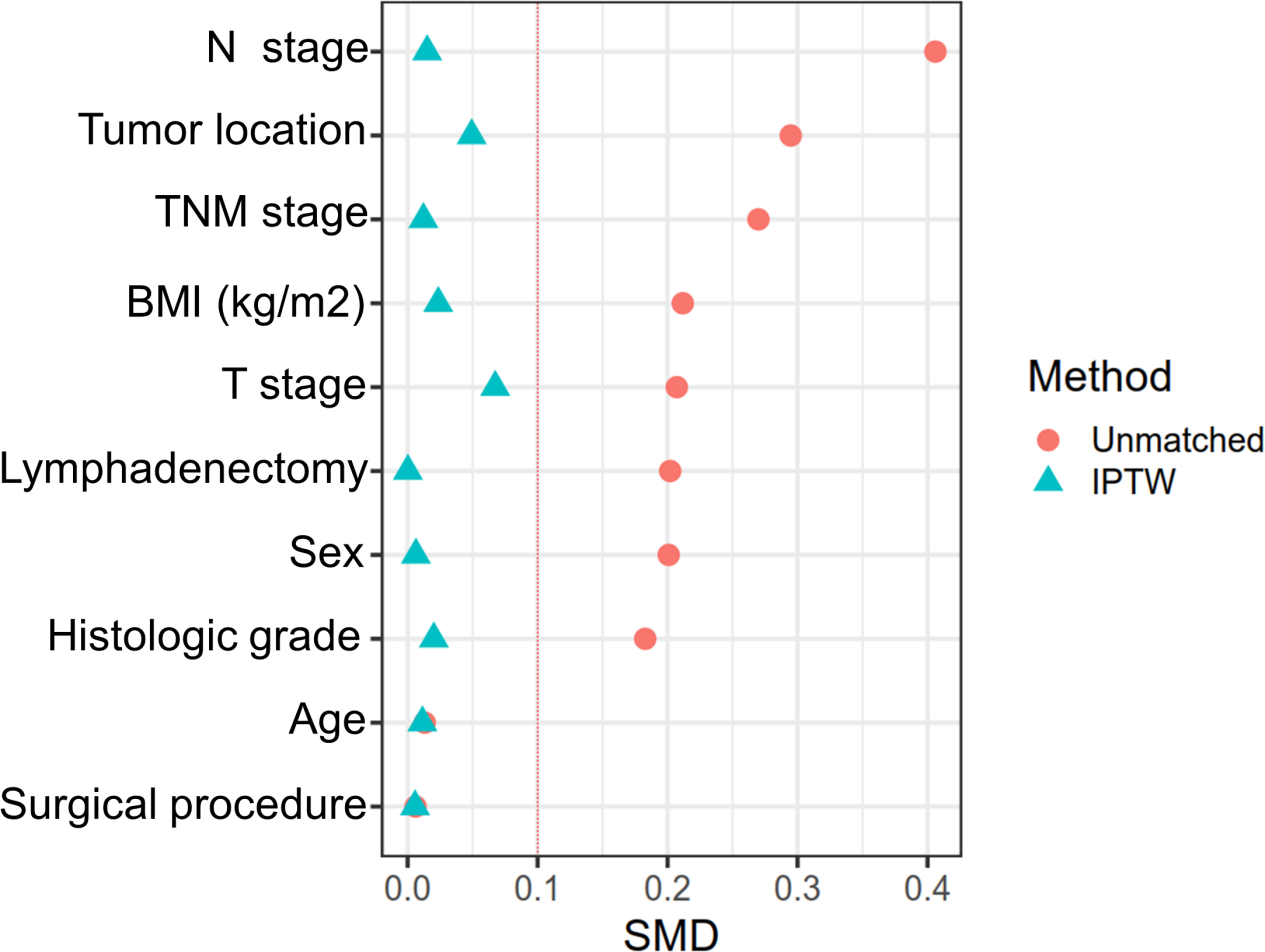
The difference between the low-ISS and high-ISS of influencing factors before matching and after IPTW.

The relationship between ISS and patients’ clinicopathological features is presented in **Table 2**. Before IPTW, the low and high ISS groups were unbalanced (SMD > 0.1) on most baseline features, including N stage, tumor location, TNM stage, BMI, T stage, lymphadenectomy, sex, and histologic grade. However, after IPTW, all baseline features were well balanced (SMD < 0.1) (**Figure 4**).

**Table 2:** The difference between low and high ISS before and after IPTW.

Characteristic	Before IPTW		P-value	After IPTW		P-value
	Low ISS	High ISS		Low ISS	High ISS	
	n=249	n=148		n=392	n=404	
Sex			0.076			0.957
Female	70 (28.1%)	29 (19.6%)		98.7 (25.2%)	100.8 (24.9%)	
Male	179 (71.9%)	119 (80.4%)		292.9 (74.8%)	303.5 (75.1%)	
Age			0.899			0.918
≤65	204 (81.9%)	122 (82.4%)		322.2 (82.3%)	334.3 (82.7%)	
>65	45 (18.1%)	26 (17.6%)		69.5 (17.7%)	69.9 (17.3%)	
BMI (kg/m2)			0.117			0.976
≤18.5	29 (11.6%)	14 (9.5%)		41.4 (10.6%)	44.1 (10.9%)	
18.5-25	183 (73.5%)	100 (67.6%)		282.4 (72.1%)	293.6 (72.6%)	
≥25	37 (14.9%)	34 (23.0%)		67.8 (17.3%)	66.6 (16.5%)	
Histologic grade			0.214			0.984
Gx/G1	96 (38.6%)	68 (45.9%)		160.0 (40.8%)	163.0 (40.3%)	
G2	137 (55.0%)	68 (45.9%)		204.3 (52.2%)	211.1 (52.2%)	
G3	16 (6.4%)	12 (8.1%)		27.4 (7.0%)	30.2 (7.5%)	
Tumor location			0.018			0.912
Proximal	23 (9.2%)	8(5.4%)		30.7 (7.8%)	36.4 (9.0%)	
Mid	168 (67.5%)	87 (58.8%)		256.5 (65.5%)	256.9 (63.5%)	
Distal	58 (23.3%)	53 (35.8%)		104.5 (26.7%)	111.0 (27.5%)	
T stage			0.279			0.948
T1	69 (27.7%)	29 (19.6%)		97.9 (25.0%)	96.0 (23.7%)	
T2	43 (17.3%)	31 (20.9%)		73.0 (18.6%)	84.9 (21.0%)	
T3	130 (52.2%)	85 (57.4%)		211.1 (53.9%)	215.4 (53.3%)	
T4a	7(2.8%)	3(2.0%)				
N stage			0.001			0.999
N0	131 (52.6%)	59 (39.9%)		189.8 (48.5%)	195.0 (48.2%)	
N1	72 (28.9%)	37 (25.0%)		110.0 (28.1%)	114.7 (28.4%)	
N2	40 (16.1%)	40 (27.0%)		75.6 (19.3%)	76.8 (19.0%)	
N3	6(2.4%)	12 (8.1%)		16.3 (4.2%)	17.8 (4.4%)	
TNM stage			0.078			1.000
I	62 (24.9%)	32 (21.6%)		94.0 (24.0%)	96.3 (23.8%)	
II	82 (32.9%)	36 (24.3%)		117.0 (29.9%)	119.3 (29.5%)	
III	98 (39.4%)	71 (48.0%)		163.2 (41.7%)	169.9 (42.0%)	
IVA	7(2.8%)	9(6.1%)		17.6 (4.5%)	18.8 (4.7%)	
Lymphadenectomy			0.065			0.998
Two-field	210 (84.3%)	113 (76.4%)		319.8 (81.7%)	330.1 (81.7%)	
Three-field	39 (15.7%)	35 (23.6%)		71.9 (18.3%)	74.1 (18.3%)	
Surgical procedure			0.953			0.959
McKeown	225 (90.4%)	134 (90.5%)		353.4 (90.2%)	365.5 (90.4%)	
Ivor Lewis	24 (9.6%)	14 (9.5%)		38.2 (9.8%)	38.8 (9.6%)	

### Survival analysis of the effect of ISS on prognosis before and after adjustment for IPTW

**Figure 5:**
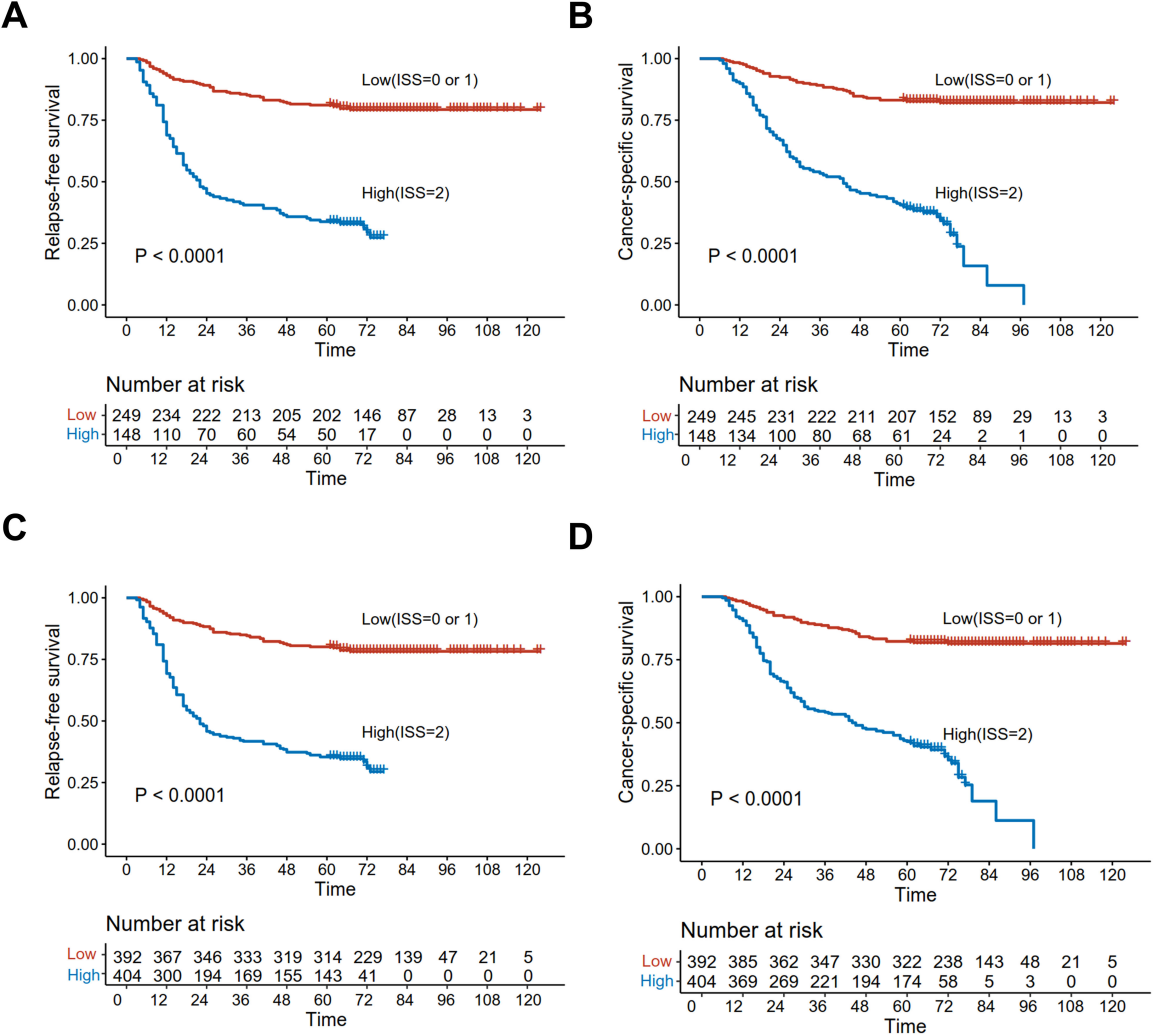
Kaplan-Meier curves analyzed the RFS and CSS of low and high ISS before matching [RFS **(A)**; CSS **(B)**] and after IPTW [RFS **(C)**; CSS **(D)**].

Before adjustment, the five-year RFS was 40.5% in cases with high ISS and 81.1% in those with low ISS, while the values for five-year CSS were 40.5 and 83.1%, respectively, with significant differences (*p < 0.001*) (**Figures 5A, B**). Whereas, after IPTW adjustment, five-year RFS were 80.1 and 35.4%, and those for five-year CSS were 82.3 and 42.5% for high and low cases, respectively, both of which were significant (*p < 0.001*) (**Figures 5C, D**).

Additionally, multivariate analysis demonstrated that before IPTW adjustment, ISS was independently predictive of adverse RFS (HR = 5.21, 95% CI: 3.62 - 7.49, *p < 0.001*) and CSS (95% CI: 3.96 - 8.47, HR = 5.79, *p < 0.001*) (**Supplementary Table S2**). Consistently, after IPTW adjustment, ISS was found to be independently predictive of both RFS (95% CI: 4.24 - 8.98, HR = 6.17, *p < 0.001*) and CSS (95% CI: 4.66 - 10.75, HR = 7.08, *p < 0.001*) (**Table 3**).

**Table 3:** Univariate and multivariate analysis of factors affecting RFS and CSS in ESCC patients after IPTW.

Characteristic	RFS				CSS			
	Univariate	P value	Multivariate	P value	Univariate	P value	Multivariate	P value
	HR (95% CI)		HR (95% CI)		HR (95% CI)		HR (95% CI)	
Sex								
Female								
Male	0.76 (0.49-1.20)	0.244			0.82 (0.52-1.31)	0.412		
Age								
≤65								
>65	1.01 (0.65-1.58)	0.955			0.94 (0.58-1.52)	0.803		
BMI (kg/m2)								
≤18.5								
18.5-25	0.83 (0.47-1.46)	0.511			0.75 (0.42-1.34)	0.328		
≥25	0.65 (0.34-1.24)	0.194			0.59 (0.30-1.16)	0.126		
Histologic grade								
Gx/G1								
G2	0.96 (0.67-1.38)	0.833			0.93 (0.65-1.34)	0.712		
G3	1.60 (0.95-2.70)	0.075			1.47 (0.84-2.57)	0.173		
Tumor location								
Proximal								
Mid	0.78 (0.38-1.58)	0.487			0.73 (0.34-1.53)	0.401		
Distal	0.90 (0.43-1.86)	0.768			0.83 (0.38-1.80)	0.643		
T stage								
T1								
T2	1.88 (0.98-3.60)	0.056	1.37 (0.71-2.63)	0.348	1.96 (1.01-3.80)	0.047	1.39 (0.72-2.67)	0.326
T3	3.28 (1.95-5.50)	<0.001	2.62 (1.51-4.56)	0.001	3.28 (1.93-5.59)	<0.001	2.59 (1.48-4.51)	0.001
T4a	5.21 (1.78-15.21)	0.003	5.43 (2.35-12.56)	<0.001	5.42 (1.77-16.57)	0.03	4.54 (1.17-17.70)	0.029
N stage								
N0								
N1	2.17 (1.40-3.38)	0.001	2.49 (1.58-3.94)	<0.001	2.17 (1.37-3.43)	0.001	2.37 (1.45-3.86)	0.001
N2	3.58 (2.35-5.46)	<0.001	2.90 (1.79-4.71)	<0.001	3.52 (2.26-5.47)	<0.001	2.90 (1.78-4.72)	<0.001
N3	3.92 (1.72-8.95)	0.001	3.73 (1.90-7.32)	<0.001	3.72 (1.71-8.09)	0.001	2.71 (1.24-5.92)	0.012
Lymphadenectomy								
Two-field								
Three-field	0.94 (0.62-1.44)	0.785			0.96 (0.63-1.46)	0.831		
Surgical procedure								
McKeown								
Ivor Lewis	0.89 (0.52-1.51)	0.666			0.88 (0.51-1.53)	0.652		
ISS								
Low								
High	4.74 (3.30-6.82	<0.001	6.17 (4.24-8.98	<0.001	5.82 (3.91-8.67)	<0.001	7.08 (4.66-10.75	<0.001

### Analysis of therapeutic effect of ESCC patients on PACT under different ISS groups

In this study, paclitaxel plus cisplatin was recommended after R0 resection in patients with postoperative pathologically confirmed T4aN0M0 or T1-4aN+M0. The effect of postoperative adjuvant chemotherapy on the prognosis of patients was then evaluated in low and high ISS subgroups. In the high ISS group, the 5-year CSS and 5-year RFS of patients who received postoperative adjuvant chemotherapy (PACT) were significantly improved compared with those who did not receive PACT (CSS:6.50% vs 39.5%, *p <* 0.001, **Supplementary Figure S4A**; RFS:2.20% vs 30.9%, *p <* 0.001, **Supplementary Figure S4B**). However, in the low ISS subgroup, the PACT did not significantly improve CSS and RFS (CSS:76.2% vs 74.1%,*p=*0.931, **Supplementary Figure S4C**; RFS:74.6% vs 72.4%,*p=* 0.678, **Supplementary Figure S4D**). These data suggest that patients with ESCC in a higher level of immunosuppressive microenvironment may be more responsive to PACT.

## Discussion

This study employed multiplex immunofluorescence assays to investigate the association between NK cell proportion, HLA-E levels, and both cumulative incidence of recurrence (CIR) and cumulative incidence of tumor-related death (CID) in ESCC patients. The immunosuppression score (ISS), derived from NK cell proportion and HLA-E expression, proved to be a more robust predictor of recurrence and tumor-related mortality compared to NK cell status or HLA-E levels alone. Moreover, after adjusting for group differences using inverse probability treatment weighting (IPTW), a high ISS was independently associated with poor recurrence-free survival (RFS) and cancer-specific survival (CSS) outcomes in patients.

The literature suggests that HLA-E expression in tumors is associated with poor outcomes in gastric, pancreatic, and breast cancer patients (19, 22, 23). Generally, HLA-E is expressed in dendritic cells, tumor cells, macrophages, and other nucleated cells within tumor nests (24, 25). Therefore, solely evaluating the HLA-E expression in tumor cells may not be sufficient; understanding its presence within the tumor microenvironment (TME) provides more compelling evidence. In this study, the level of HLA-E expression in ESCC tumor nests was quantified, and immunoreactivity scores (IRS) were assessed. The results indicated that elevated HLA-E levels (IRS ≥ 6) are associated with lymph node metastasis in ESCC and are predictive of both the cumulative incidence of recurrence (CIR) and tumor-related death (CID).

Immune system cells, including NK cells, have been identified as potential biomarkers for predicting the prognosis of cancer patients (26–29). NK cells, as innate immune effectors, play a critical role in the anti-tumor immune response within the TME. Reduced NK cell infiltration has been linked to disease progression and shorter survival in cancer patients (16, 17). NK cells are characterized by CD3 negative and CD56 positive expression, which can be divided into CD56 bright and CD56 dim subgroups (19). In this study, a multiplex immunofluorescence assay was used to evaluate the expression of CD56 and CD3, confirming that the status of NK cells significantly influences recurrence and tumor-related mortality in ESCC patients.

Recent research has demonstrated that combining PD-L1 expression with tumor-infiltrating lymphocytes (TILs) can characterize four distinct TME types. Tumors positive for both PD-L1 and TILs are more likely to respond to immune checkpoint blockade (ICB) therapy (29). Moreover, stratifying patients based on PD-L1 expression and TIL status further differentiate overall survival in esophageal cancer patients (30). A study in gastric cancer also indicated that NK cell status, combined with HLA-E expression, can serve as a prognostic factor for evaluating recurrence-free survival (RFS) in advanced gastric cancer patients (19). Based on NK cell proportion and HLA-E expression, an immunosuppression score (ISS) was proposed as a biomarker to evaluate the immune status of the TME. This study found significant differences in recurrence and tumor-related mortality between high and low ISS groups. Notably, after adjusting for baseline differences using inverse probability treatment weighting (IPTW), ISS was independently predictive of poor cancer-specific survival (CSS) and RFS. This may be attributed to the immunosuppressive effects of high HLA-E expression and decreased NK cell infiltration in the TME, which together contribute to immune escape and poor oncological outcomes.

The limitations of this study include its retrospective nature, based on data from ESCC patients who underwent surgical resection at two centers. Further prospective research is needed to validate the prognostic value of HLA-E expression and NK cell status. Additionally, while this study focused on ESCC confirmed by histopathology, the prognostic significance of NK cell status and HLA-E expression in esophageal adenocarcinoma remains unclear. However, this is the first study to combine NK cell status and HLA-E expression to propose the ISS as a marker of immune status in the TME, specifically for ESCC.

In conclusion, this study links NK cell status and HLA-E expression with poor RFS and CSS in ESCC patients. The ISS, which integrates both parameters, effectively stratifies patients into distinct prognostic subgroups. After IPTW adjustment for confounding factors, ISS emerged as a key prognostic factor for ESCC. Given the limited therapeutic value of current PD-L1 pathway inhibitors, future research should explore adoptive NK cell therapies or strategies targeting the NKG2A/HLA-E axis for improved therapeutic outcomes.

## Data Availability

The raw data supporting the conclusions of this article will be made available by the authors, without undue reservation.

## Supplementary material

Supplementary Figure S1 The IRS of HLA-E **(A)** and NK cell proportion **(B)** in the ESCC patient population.

Supplementary Figure S2 HLA-E expression and NK cell status calculation of ISS.

Supplementary Figure S3 The ROC curve compared the value of HLA-E expression, NK cell status, and ISS in predicting patient recurrence **(A)** and tumor-related death **(B)**.

Supplementary Figure S4 Kaplan-Meier curves analyzed the CSS and RFS of ESCC patients in low and high ISS group.
