# Supplementary figures and images for: Prognostic value of immunosuppression scores in patients with esophageal squamous cell carcinoma: a multicenter study
Source: Front Immunol. 2025 Jan 7;15:1517968. doi: 10.3389/fimmu.2024.1517968 (PMC11752912; doi:10.3389/fimmu.2024.1517968)

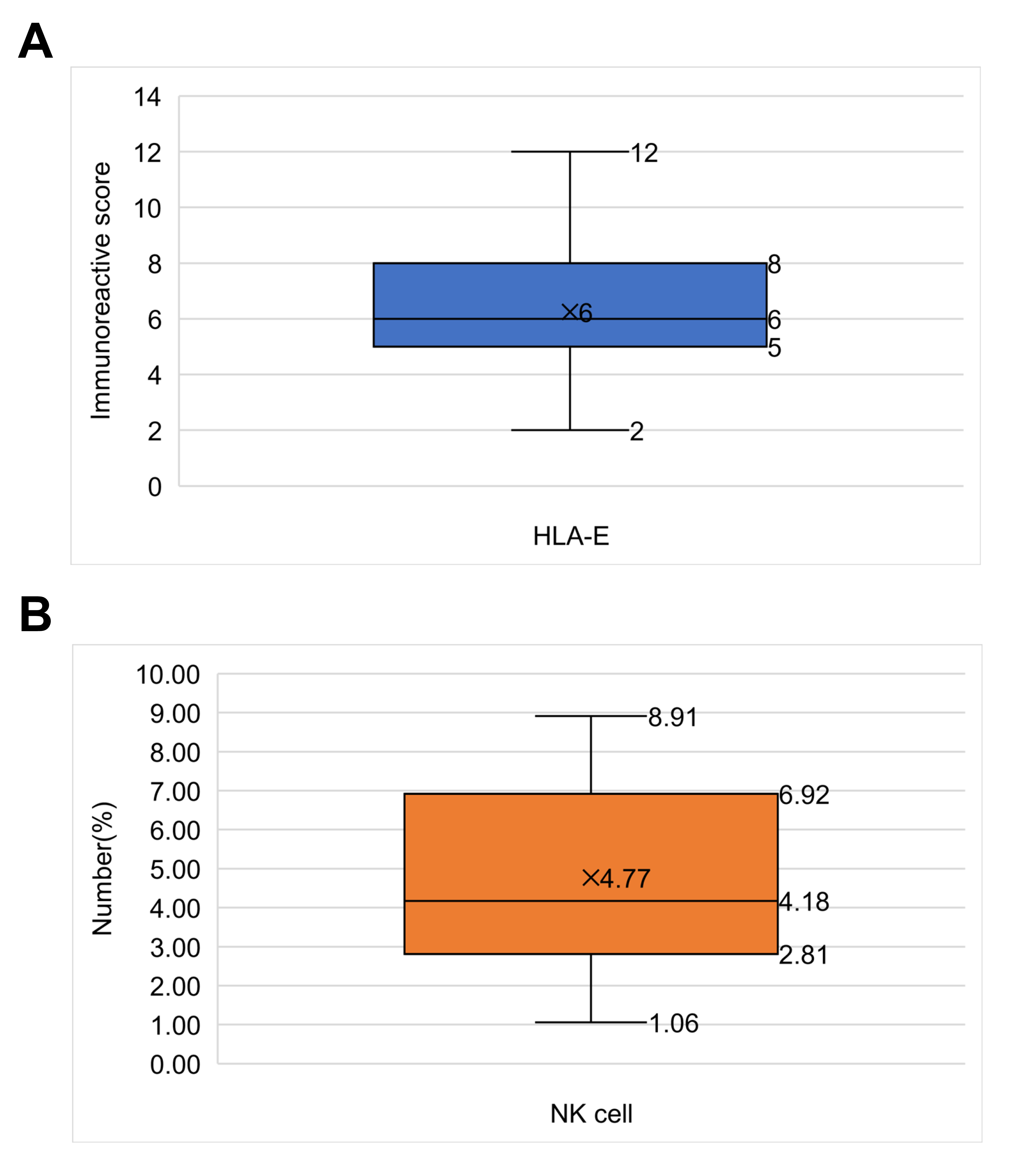

Supplement: Supplementary Figure S1 — The IRS of HLA-E (A) and NK cell proportion (B) in the ESCC patient population. [file Image1.tif]

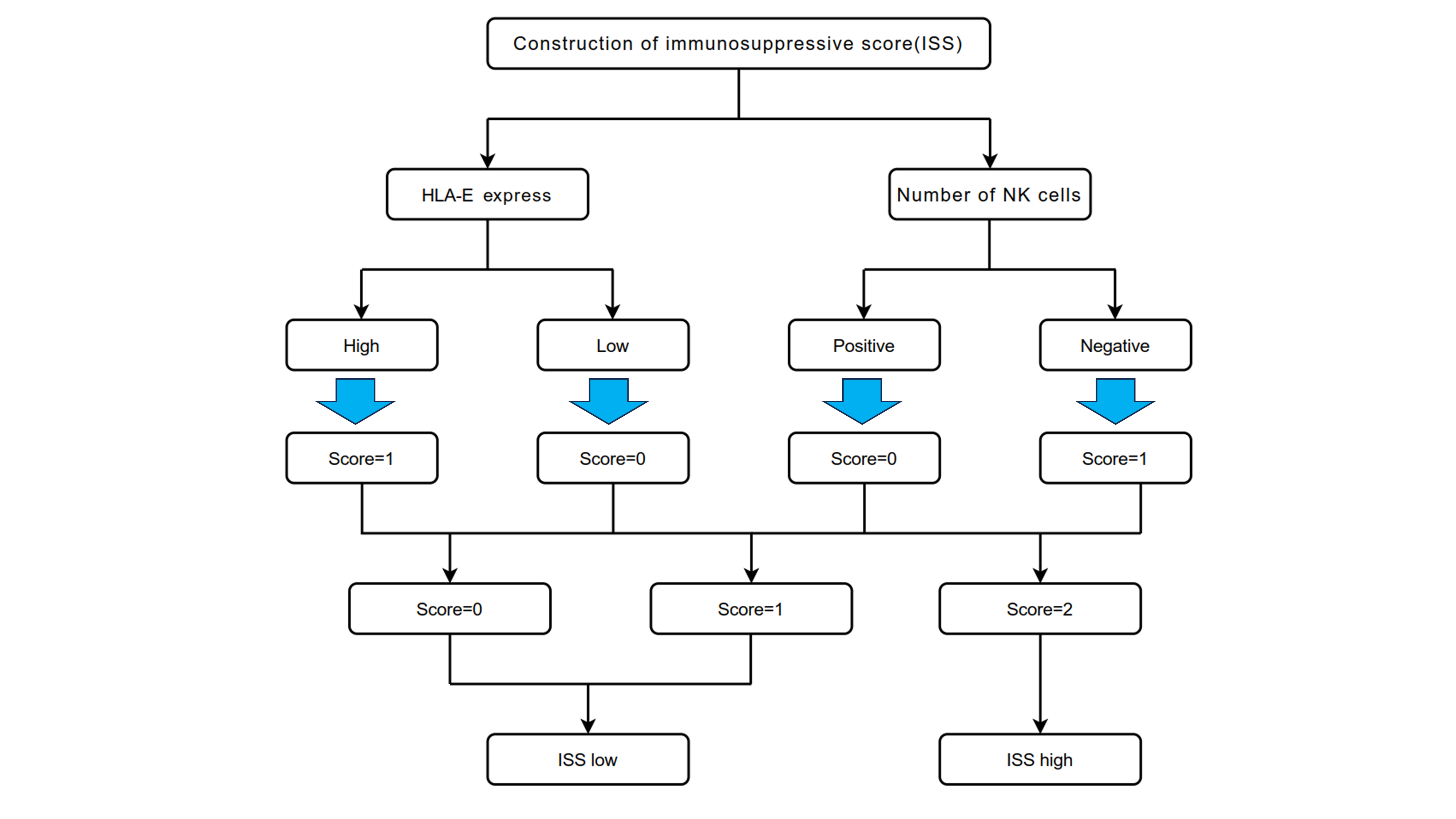

Supplement: Supplementary Figure S2 — HLA-E expression and NK cell status calculation of ISS. [file Image2.tif]

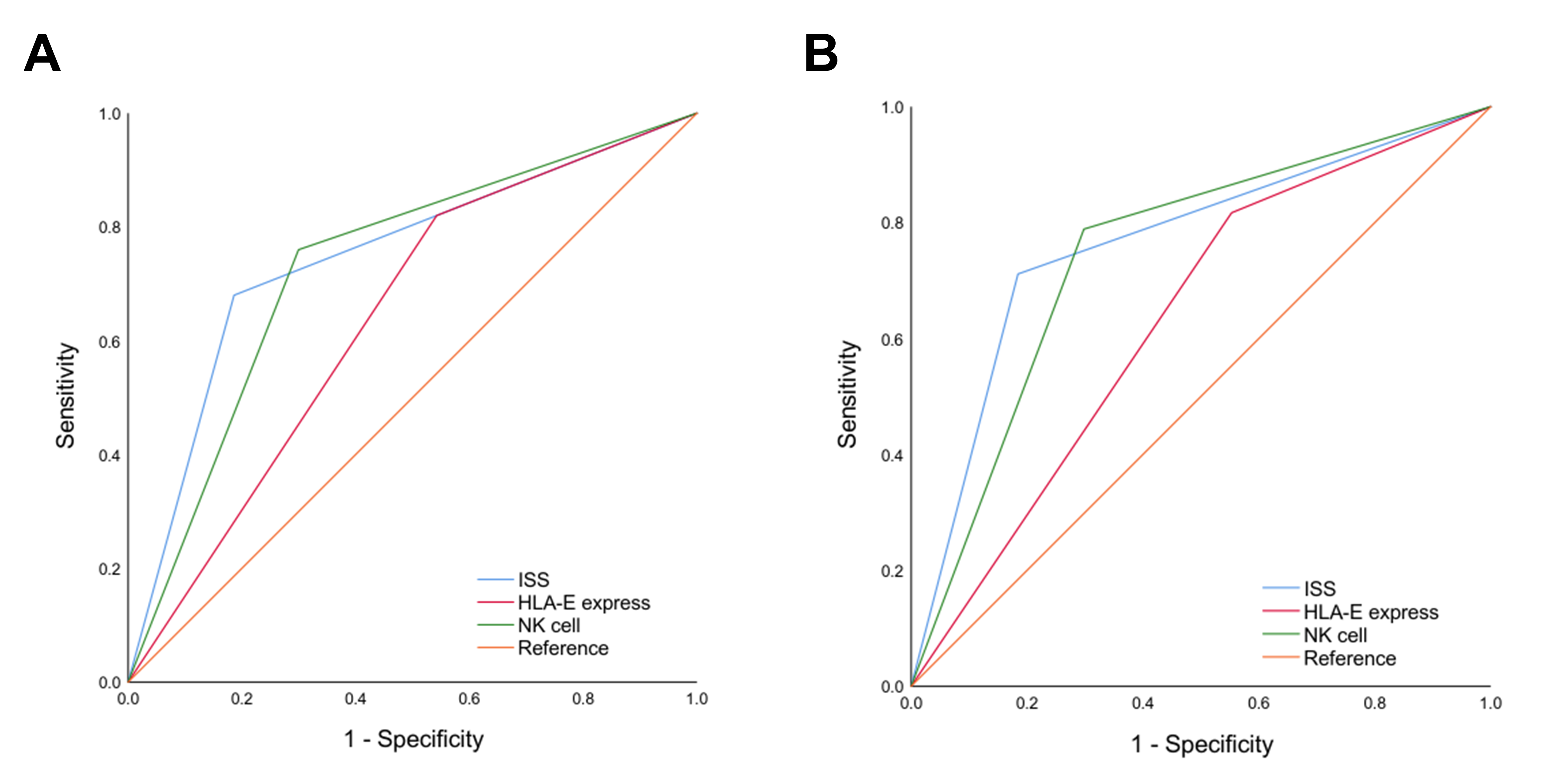

Supplement: Supplementary Figure S3 — The ROC curve compared the value of HLA-E expression, NK cell status, and ISS in predicting patient recurrence (A) and tumor-related death (B). [file Image3.tif]

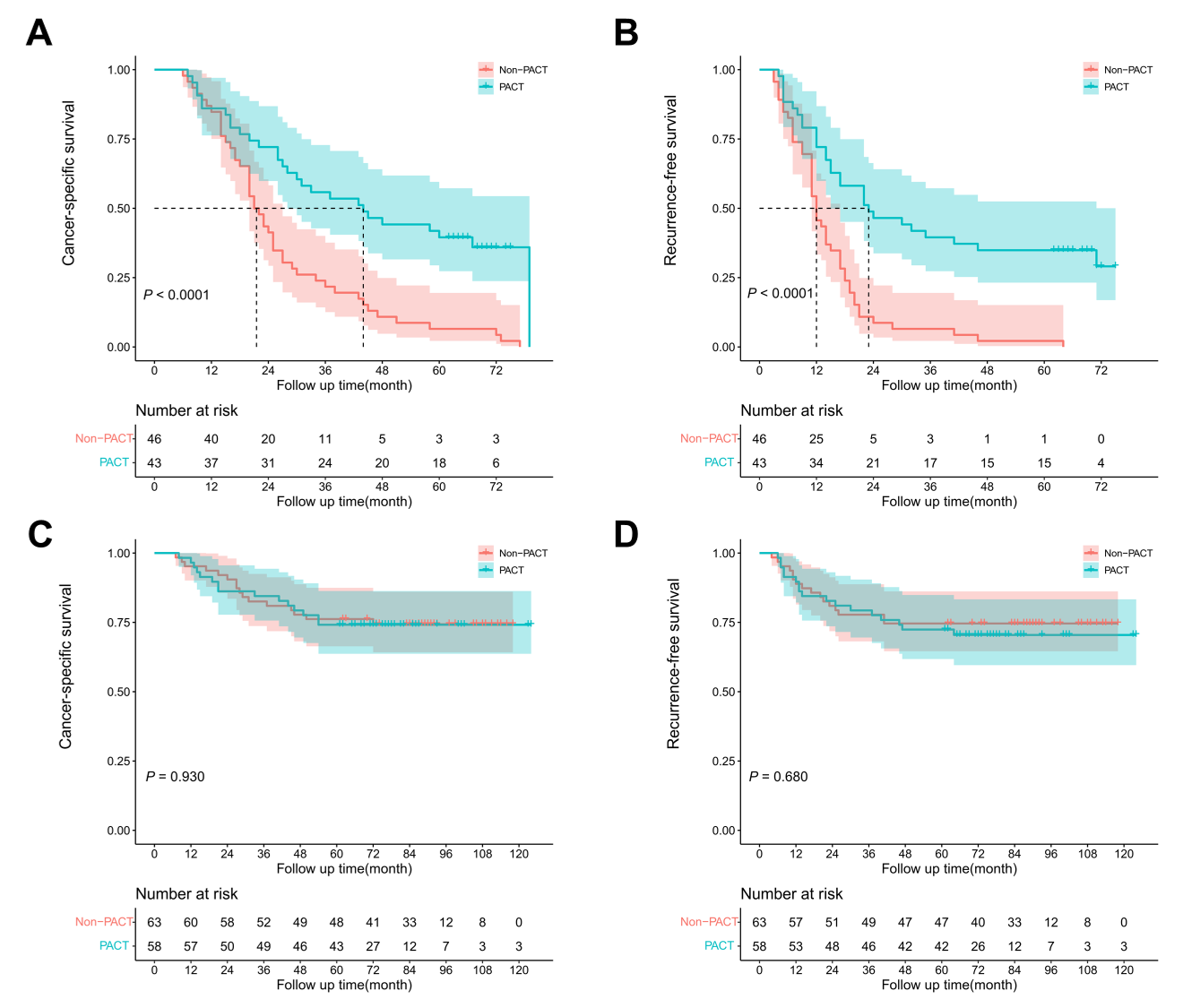

Supplement: Supplementary Figure S4 — Kaplan-Meier curves analyzed the CSS and RFS of ESCC patients in low and high ISS group. [file Image4.tif]
