# Supplementary material for: Prognostic value of immunosuppression scores in patients with esophageal squamous cell carcinoma: a multicenter study
Source: Front Immunol. 2025 Jan 7;15:1517968. doi: 10.3389/fimmu.2024.1517968 (PMC11752912; doi:10.3389/fimmu.2024.1517968)
Supplement: Supplementary file 5 [file Table1.docx]

**Table S1** Univariate and multivariate analysis the effects of HLA-E expression and NK cell status on RFS and CSS in ESCC patients

| Characteristic | |  | RFS |  |  |  |  |  |  | CSS |  |  |  |  |  |
| --- | --- | --- | --- | --- | --- | --- | --- | --- | --- | --- | --- | --- | --- | --- | --- |
|  |  |  | Univariate | |  | Multivariate | |  |  | Univariate | |  | Multivariate | |  |
|  |  |  | HR (95% CI) | | P value | HR (95% CI) | | P value |  | HR (95% CI) | | P value | HR (95% CI) | | P value |
| Sex |  |  |  |  |  |  |  |  |  |  |  |  |  |  |  |
| Female |  |  |  |  |  |  |  |  |  |  |  |  |  |  |  |
| Male |  |  | 0.656(0.438-0.984) | | 0.041 | 1.177(0.763-1.815) | | 0.462 |  | 0.670(0.441-1.018) | | 0.060 |  |  |  |
| Age |  |  |  |  |  |  |  |  |  |  |  |  |  |  |  |
| ≤65 |  |  |  |  |  |  |  |  |  |  |  |  |  |  |  |
| ＞65 |  |  | 1.055(0.700-1.589) | | 0.799 |  |  |  |  | 1.001(0.651-1.541) | | 0.996 |  |  |  |
| BMI (kg/m2) | |  |  |  |  |  |  |  |  |  |  |  |  |  |  |
| ≤18.5 |  |  |  |  |  |  |  |  |  |  |  |  |  |  |  |
| 18.5-25 |  |  | 0.925(0.555-1.543) | | 0.766 |  |  |  |  | 0.858(0.513-1.583) | | 0.559 |  |  |  |
| ≥25 |  |  | 0.937(0.513-1.711) | | 0.831 |  |  |  |  | 0.859(0.466-1.583) | | 0.625 |  |  |  |
| Histologic grade | |  |  |  |  |  |  |  |  |  |  |  |  |  |  |
| Gx/G1 |  |  |  |  |  |  |  |  |  |  |  |  |  |  |  |
| G2 |  |  | 0.919(0.658-1.285) | | 0.623 |  |  |  |  | 0.900(0.640-1.267) | | 0.547 |  |  |  |
| G3 |  |  | 1.518(0.865-2.664) | | 0.146 |  |  |  |  | 1.355(0.745-2.463) | | 0.320 |  |  |  |
| Tumor location | |  |  |  |  |  |  |  |  |  |  |  |  |  |  |
| Proximal |  |  |  |  |  |  |  |  |  |  |  |  |  |  |  |
| Mid |  |  | 0.935(0.513-1.707) | | 0.828 |  |  |  |  | 0.951(0.508-1.781) | | 0.876 |  |  |  |
| Distal |  |  | 1.169(0.622-2.197) | | 0.628 |  |  |  |  | 1.176(0.609-2.272) | | 0.629 |  |  |  |
| T stage |  |  |  |  |  |  |  |  |  |  |  |  |  |  |  |
| T1 |  |  |  |  |  |  |  |  |  |  |  |  |  |  |  |
| T2 |  |  | 1.901(1.032-3.503) | | 0.039 | 1.154(0.614-2.169) | | 0.655 |  | 2.030(1.072-3.843) | | 0.030 | 1.227(0.636-2.364) | | 0.542 |
| T3 |  |  | 3.494(2.119-5.761) | | ＜0.001 | 2.350(1.368-4.038) | | 0.002 |  | 3.689(2.176-6.254) | | ＜0.001 | 2.339(1.330-4.113) | | 0.003 |
| T4a |  |  | 4.085(1.515-11.010) | | 0.005 | 3.907(1.407-10.853) | | 0.009 |  | 4.352(1.594-11.883) | | 0.004 | 2.786(0.942-8.238) | | 0.064 |
| N stage |  |  |  |  |  |  |  |  |  |  |  |  |  |  |  |
| N0 |  |  |  |  |  |  |  |  |  |  |  |  |  |  |  |
| N1 |  |  | 1.694(1.103-2.601) | | 0.160 | 1.902(1.212-2.984) | | 0.005 |  | 1.810(1.161-2.822) | | 0.009 | 1.913(1.210-3.026) | | 0.006 |
| N2 |  |  | 4.467(3.005-6.640) | | ＜0.001 | 2.891(1.882-4.442) | | ＜0.001 |  | 4.646(3.075-7.019) | | ＜0.001 | 2.925(1.875-4.564) | | ＜0.001 |
| N3 |  |  | 6.686(3.653-12.235) | | ＜0.001 | 3.715(1.953-7.065) | | ＜0.001 |  | 6.467(3.510-11.915) | | ＜0.001 | 2.972(1.529-5.777) | | 0.001 |
| Lymphadenectomy | |  |  |  |  |  |  |  |  |  |  |  |  |  |  |
| Two-field |  |  |  |  |  |  |  |  |  |  |  |  |  |  |  |
| Three-field | |  | 1.171(0.789-1.737) | | 0.433 |  |  |  |  | 1.197(0.801-1.790) | | 0.380 |  |  |  |
| Surgical procedure | |  |  |  |  |  |  |  |  |  |  |  |  |  |  |
| McKeown | |  |  |  |  |  |  |  |  |  |  |  |  |  |  |
| Ivor Lewis | |  | 0.792(0.448-1.398) | | 0.420 |  |  |  |  | 0.748(0.414-1.352) | | 0.337 |  |  |  |
| HLA-E |  |  |  |  |  |  |  |  |  |  |  |  |  |  |  |
| Low |  |  |  |  |  |  |  |  |  |  |  |  |  |  |  |
| High |  |  | 2.711(1.818-4.044) | | ＜0.001 | 1.968(1.291-3.000) | | 0.002 |  | 2.628(1.747-3.953) | | ＜0.001 | 1.725(1.126-2.643) | | 0.012 |
| NK cell |  |  |  |  |  |  |  |  |  |  |  |  |  |  |  |
| Low |  |  |  |  |  |  |  |  |  |  |  |  |  |  |  |
| High |  |  | 5.382(3.695-7.841) | | ＜0.001 | 4.606(3.106-6.832) | | ＜0.001 |  | 6.687(4.425-10.107) | | ＜0.001 | 5.681(3.693-8.739) | | ＜0.001 |
