# Supplementary material for: Prognostic value of immunosuppression scores in patients with esophageal squamous cell carcinoma: a multicenter study
Source: Front Immunol. 2025 Jan 7;15:1517968. doi: 10.3389/fimmu.2024.1517968 (PMC11752912; doi:10.3389/fimmu.2024.1517968)
Supplement: Supplementary file 6 [file Table2.docx]

**Table S2** Univariate and multivariate analysis of factors affecting RFS and CSS in ESCC patients before IPTW

| Characteristic | | RFS |  |  |  |  |  | CSS |  |  |  |  |  |
| --- | --- | --- | --- | --- | --- | --- | --- | --- | --- | --- | --- | --- | --- |
|  |  | Univariate | |  | Multivariate | |  | Univariate | |  | Multivariate | |  |
|  |  | HR (95% CI) | | P value | HR (95% CI) | | P value | HR (95% CI) | | P value | HR (95% CI) | | P value |
| Sex |  |  |  |  |  |  |  |  |  |  |  |  |  |
| Female |  |  |  |  |  |  |  |  |  |  |  |  |  |
| Male |  | 0.656(0.438-0.984) | | 0.041 | 1.23(0.80-1.89) | | 0.343 |  |  |  |  |  |  |
| Age |  |  |  |  |  |  |  |  |  |  |  |  |  |
| ≤65 |  |  |  |  |  |  |  |  |  |  |  |  |  |
| ＞65 |  | 1.055(0.700-1.589) | | 0.799 |  |  |  |  |  |  |  |  |  |
| BMI (kg/m2) | |  |  |  |  |  |  |  |  |  |  |  |  |
| ≤18.5 |  |  |  |  |  |  |  |  |  |  |  |  |  |
| 18.5-25 |  | 0.925(0.555-1.543) | | 0.766 |  |  |  |  |  |  |  |  |  |
| ≥25 |  | 0.937(0.513-1.711) | | 0.831 |  |  |  |  |  |  |  |  |  |
| Histologic grade | |  |  |  |  |  |  |  |  |  |  |  |  |
| Gx/G1 |  |  |  |  |  |  |  |  |  |  |  |  |  |
| G2 |  | 0.919(0.658-1.285) | | 0.623 |  |  |  |  |  |  |  |  |  |
| G3 |  | 1.518(0.865-2.664) | | 0.146 |  |  |  |  |  |  |  |  |  |
| Tumor location | |  |  |  |  |  |  |  |  |  |  |  |  |
| Proximal |  |  |  |  |  |  |  |  |  |  |  |  |  |
| Mid |  | 0.935(0.513-1.707) | | 0.828 |  |  |  |  |  |  |  |  |  |
| Distal |  | 1.169(0.622-2.197) | | 0.628 |  |  |  |  |  |  |  |  |  |
| T stage |  |  |  |  |  |  |  |  |  |  |  |  |  |
| T1 |  |  |  |  |  |  |  |  |  |  |  |  |  |
| T2 |  | 1.901(1.032-3.503) | | 0.039 | 1.20(0.64-2.25) | | 0.572 |  |  |  | 1.27 (0.66-2.44 | | 0.480 |
| T3 |  | 3.494(2.119-5.761) | | ＜0.001 | 2.49(1.45-4.27) | | 0.001 |  |  |  | 2.47 (1.41-4.34 | | 0.002 |
| T4a |  | 4.085(1.515-11.010) | | 0.005 | 3.98(1.42-11.12) | | 0.009 |  |  |  | 2.73 (0.92-8.16 | | 0.071 |
| N stage |  |  |  |  |  |  |  |  |  |  |  |  |  |
| N0 |  |  |  |  |  |  |  |  |  |  |  |  |  |
| N1 |  | 1.694(1.103-2.601) | | 0.160 | 1.81(1.16-2.83) | | 0.009 |  |  |  | 1.82(1.15-2.87) | | 0.010 |
| N2 |  | 4.467(3.005-6.640) | | ＜0.001 | 2.80(1.83-4.29) | | ＜0.001 |  |  |  | 2.77(1.78-4.31) | | ＜0.001 |
| N3 |  | 6.686(3.653-12.235) | | ＜0.001 | 3.99(2.09-7.59) | | ＜0.001 |  |  |  | 3.02(1.54-5.90) | | ＜0.001 |
| Lymphadenectomy | |  |  |  |  |  |  |  |  |  |  |  |  |
| Two-field |  |  |  |  |  |  |  |  |  |  |  |  |  |
| Three-field | | 1.171(0.789-1.737) | | 0.433 |  |  |  |  |  |  |  |  |  |
| Surgical procedure | |  |  |  |  |  |  |  |  |  |  |  |  |
| McKeown | |  |  |  |  |  |  |  |  |  |  |  |  |
| Ivor Lewis | | 0.792(0.448-1.398) | | 0.420 |  |  |  |  |  |  |  |  |  |
| ISS |  |  |  |  |  |  |  |  |  |  |  |  |  |
| Low |  |  |  |  |  |  |  |  |  |  |  |  |  |
| High |  | 5.28(3.76-7.42) | | ＜0.001 | 5.21(3.62-7.49) | | ＜0.001 | 6.21(4.31-8.95) |  | ＜0.001 | 5.79(3.96-8.47) | | ＜0.001 |
